# Exploring the contexts, mechanisms and outcomes of a torture, abuse and dental anxiety service in Norway: a realist evaluation

**DOI:** 10.1186/s12913-022-07913-7

**Published:** 2022-04-22

**Authors:** Emilie Bryne, Sarah Catherine Patricia Duff Hean, Kjersti Berge Evensen, Vibeke Hervik Bull

**Affiliations:** 1 Oral Health Centre of Expertise Rogaland, Torgveien 21 B, 4016 Stavanger, Norway; 2 Faculty of Social Sciences, University of Stavanger, Postboks 8600 Forus, 4036 Stavanger, Norway

**Keywords:** Dental anxiety, Dental phobia, Torture, Abuse, Oral health, Dental health services, Health services, Oral health policy

## Abstract

**Background:**

Torture, abuse and dental anxiety (TADA) are often precursors to developing a pathological relationship with dental care due to elevated anxiety. Consequently, patients who suffer from one or more of these tend to avoid dental services. This could leave them with severe tooth decay, which could affect their general and psychosocial health. Norwegian dental services have implemented the TADA service to specifically alleviate dental anxiety and restore oral health for the TADA patient group. However, the service has not been evaluated, and there is a need to understand how and why this service works, for whom, under what circumstances. Therefore, this study aimed to develop theories on how the service’s structure alleviates dental anxiety and restores these patients’ oral health. Although developed in a Norwegian context, these theories may be applicable to other national and international contexts.

**Methods:**

This realist evaluation comprised multiple sequential methods of service and policy documents (*n* = 13), followed by interviews with service developers (*n* = 12).

**Results:**

The analysis suggests that, by subsidising the TADA service, the Norwegian state has removed financial barriers for patients. This has improved their access to the service and, hence, their service uptake. National guidelines on service delivery are perceived as open to interpretation, and can hereby meet the needs of a heterogeneous patient group. The services have become tailored according to the available regional resources and heterogeneous needs of the patient population. A perceived lack of explicit national leadership and cooperative practices has resulted in regional service teams becoming self-reliant and insular. While this has led to cohesion within each regional service, it is not conducive to interservice collaborations. Lastly, the complexity of migration processes and poor dissemination practices is presumed to be the cause of the lack of recruitment of torture survivors to the service.

**Conclusions:**

Policy documents and service developers described the TADA service as a hybrid bottom-up/top-down service that allows teams to practise discretion and tailor their approach to meet individual needs. Being free of charge has improved access to the service by vulnerable groups, but the service still struggles to reach torture survivors.

## Background

People subjected to torture or abuse or who have dental phobia may avoid dental services and examinations due to anxiety or the psychological triggers that the dental setting may stimulate [1–5]. With an ongoing avoidance behaviour of dental services, a fear of ‘revealing’ their mouth may emerge, leading to deteriorated oral health. This often requires dental services, such as multiple restoration and extractions. The negative cycle that may emerge from avoiding dental services can be both painful and costly for the individual and society as a whole [6–8].

### The TADA service

The Norwegian government developed the torture, abuse and dental anxiety (TADA) service in 2010 to address the challenges faced by patients who have survived torture or abuse or who have a dental phobia in their use of dental services [9]. The TADA service is unique in its bi-dimensional approach to targeting both dental anxiety and avoidance behaviour and to restoring patients’ oral health. A working group consisting of dental practitioners, psychologists and researchers was established to design and implement a dental service catering to these patients [9, 10], and cognitive behavioural therapy (CBT) was chosen as the main form of treatment for the TADA service. Both national and international research suggests that elements of CBT, specifically *in vivo* exposure therapy, could effectively treat the anxiety aspect, which is assumed to be the precursor to avoiding dental services [11–13]. The underlying assumption was that their oral health could be restored by relieving patients’ dental anxiety through CBT. The TADA service aims to deliver an equitable, standardised service to adults (> 18 years) across Norway. However, in Norway, oral health falls under county-level administration. Therefore, each county is responsible for the logistics of service and resource provision.

Patients who are eligible for the TADA service include those with a history of torture with direct consequences for their mouth, neck or head regions. A notable aspect of this patient group is that torture survivors often display comorbidities and likely struggle with post-traumatic stress disorder (PTSD) [14]. As of 2019, approximately 10,000–35,000 torture survivors resided in Norway [15]. Furthermore, patients whose dental behaviours have been impacted by sexual, physical or psychological abuse are eligible for the TADA service. These patients are included in the service due to the elevated risk of developing anxiety in the dental setting. Norwegian national reports reveal that 34% of women and 11% of men have been subjected to sexual abuse and that 1 in 20 children and adolescents have been assaulted [16, 17]. Finally, patients, who meet the Diagnostic and Statistical Manual of Mental Disorders-IV (DSM-IV) criteria for suffering from a specific phobia, are eligible for TADA services. The DSM-IV criteria are unreasonable fear, immediate anxiety response and avoidance behaviour or extreme distress, and these symptoms must have a significant impact on the patient’s life, must last for at least six months and not be caused by any other disorder [18]. Only one study within the Norwegian context has described the prevalence of dental phobia, and it revealed that 8% of 18-year-olds suffer from this [19].

From here on, the paper uses the word *dental anxiety* to express the challenges of all three patient groups because this is the common denominator for all these patients enrolled in the TADA service whatever the cause. This means that patients with a history of torture or abuse do not need to meet the DSM criteria for a dental phobia to be included in the TADA service. However, if there is no history of torture or abuse, dental phobia, as defined by the DSM, is required.

The TADA service is designed such that, before oral restoration takes place, the anxiety aspect experienced by patients is addressed first. The teams that focus on treating this anxiety through CBT are defined as TADA teams. These teams consist of psychologists and dental practitioners (dentists and dental hygienists). The psychologists assess patients’ service eligibility and train dental practitioners on how to deliver CBT. In this way, the service ensures that psychological assessment tools are appropriately used during the service assessment and delivery.

Since early 2011, the TADA service has been gradually implemented as a public service free of charge to eligible patients across Norway [9]. The service’s budget has increased yearly, starting at 2.5 million kroners in 2011 to 85 million kroners (equivalent to 8 million euros) in 2020. The budget includes the TADA teams’ salaries and its rise can be attributable to the increase of teams nationally. Currently, 52 TADA teams are in place across Norway. The year-long national waiting list reflects the high demand for the TADA service. As of 2018, 1186 patients had either completed or were undergoing dental treatment as part of the TADA service.

However, service outcomes have not yet been evaluated and remain poorly understood. How, for example, is the TADA service structured to cater for such a heterogeneous patient group? The current study addresses this question through a realist evaluation to understand *how the TADA service works, for whom, under which circumstances, and why*. In this paper, we report the findings of the evaluation in terms of the contextual elements, triggering mechanisms and resulting outcomes of the TADA service at a structural level. Although the TADA service is a Norwegian intervention, knowledge of how it is structured and how these structures function in this national context and with this vulnerable patient group, can be transferred to the international setting. These settings also face similar challenges of dental anxiety and seek to understand how treatment and dental health services for patients with a history of torture or abuse or a diagnosis of dental phobia may be set up.

Our findings related to other dimensions of the evaluation (the role that dental practitioners play in delivering the service and how the service has impacted patients) have been reported elsewhere [20, 21].

## Methods

### Realist evaluation methodology

To explore the structural workings of the TADA service, we used a realist evaluation methodology [22]. Intrinsic to this methodology was the exploration of for whom and under which circumstances the TADA service is effective and the assessment of its complex programmes and services [23, 24]. This complexity is inherent in the service’s interdisciplinary approach and diverse patient population. A realist evaluation is well suited in this regard, as it allows the researcher to more fully describe the working mechanisms of complex systems. Indeed, realist evaluations are becoming increasingly popular for addressing the complexities of health services [22, 24, 25]. Realist evaluations are underpinned by the philosophy of scientific realism, which claims that generative causal mechanisms are triggered by contextual elements that, in turn, produce observable outcomes [22, 24, 26, 27]. This philosophy distinguishes realist evaluations from other types of theory-driven approaches, as it generates data-derived insights with ontological depth. This permits valid explanations for why a given phenomenon works, for whom and under which circumstances [22, 26].

A realist evaluation begins by seeking to explain how a phenomenon like the TADA programme contributes to substantive changes in treatment outcomes [28, 29]. The assumption is that how the service leaders have developed the service was guided by one or more theories. Service developers either implicitly or explicitly rely on these theories when executing the service in specific contexts and circumstances [22]. A vital goal of the realist evaluation is, therefore, to articulate these *programme theories* – namely, to provide plausible explanations for the nature and characteristics of a phenomenon by describing its outcomes, the mechanisms responsible for these outcomes and how these mechanisms are triggered in a given contextual setting [24, 30]. Table 1 presents more precise definitions of outcomes, mechanisms and contexts, in reverse order.

**Table 1:** Definitions and reflections on contexts, mechanisms and outcomes

**Contexts (C)**: These describe the elements and background factors that allow mechanisms to be triggered. Contexts are not limited to locations but also refer to characteristics of individual service developers, deliverers and patients. It refers also to interrelationships between actors and institutional settings and their placement within wider infrastructural settings. To understand contexts, the researcher should ask: *What conditional elements or contextual components must be present for a mechanism to be triggered?* **Mechanisms (M)**: A mechanism will be triggered, if the context is conducive to this. This conception of mechanisms has two main features: resources and reasoning. The assumption here is that if certain service resources are introduced to a specific context, they will generate changes in the actors’ reasoning. In this study, these actors are service developers, deliverers, and patients. Pairing resources with reasoning defines the mechanism. Therefore, the following question is asked to reveal mechanisms: *How do the resources provided by the service impact the service deliverers, and on what assumptions, values, and beliefs do service users rely on when interacting with these resources? What is being triggered in the service, and to what particular outcome does it lead?* **Outcomes (O)**: Outcomes describe the visible output or impact the mechanisms lead to. These outcomes can be immediate, intermediate, or long term. Therefore, in analysing outcomes, the researcher should ask: *to what end does the triggered mechanism lead and what are the resulting outcomes of the triggered mechanisms in the right context?* **Context–mechanisms–outcome configurations (CMOCs)**: Generative explanations for the observed outcomes can be produced by heuristically configuring or combining contexts (C), mechanisms (M) and outcomes (O). These CMOCs provide a causative explanation for either the working of the entire service or for specific features of the service.	

The realist evaluation method deconstructs a phenomenon – in this study, the TADA service – in terms of contexts, mechanisms and outcomes. It then *re*constructs these factors in a series of context-mechanisms-outcome configurations (CMOCs). These configurations of contexts, mechanisms and outcomes yield a proposition about how the phenomenon works, for whom and under which circumstances [22, 25, 31]. In principle, the process involves explicitly making foundational assumptions about how the phenomenon (i.e., the service/programme) should work before systematically collecting evidence to test and refine or refute this theory [22, 25, 31]. Consequently, and circularly, the evaluation process starts with a theory and culminates with a theory. The whole process is, by nature, iterative and retroductive, aiming to elicit the underlying mechanisms believed to reside at an ontological depth.

### Data collection

This study employed a multi-method study design to generate the programme theories with two primary data sources: service and policy documents and interviews [32–34]. Consistent with a sequential, multi-method design [35], the analysis of the policy documents preceded the interviews and, thereby, informed the structure and foci of the interview schedule.

In a realist evaluation, existing literature in the field can be an extra source of data when developing programme theories. However, in the present study, the results of a literature search on dental services aimed at TADA patients, showed the evidence to be limited. Previous research that has studied the oral status and anxiety levels of these patient groups [14, 36–43] focuses on patient outcomes and ignores the functioning of services that provide treatment for these patients. Our study redresses this shortfall.

#### Document analysis

A search of service databases and interviews with stakeholders generated a sample of 13 policy documents and service-related grey literature (Table 2). Reviewing these documents provided a historical lens through which to understand why and how the TADA service currently operates and intends to achieve its objectives. The documents also provide descriptions of the key service dimensions, including the desired therapy component (i.e., CBT).

**Table 2:** Document analyses

No.	Title (translated into English)	Author/Year	Document type	Description
1	*Practitioners Handbook*	Myran, L., Johnsen, I.B., Årøen Lie, J.P., June 2019	Handbook	This handbook provides details on how practitioners should meet and work with the patient group. Details are provided regarding the aetiology of anxiety, and symptoms of dental phobia. Cognitive behavioural therapy and communication methods aimed at enhancing relationship building are elaborated in this handbook.
2	*Practitioners Guidance*	TADA, December 2018	Guidelines on operating practice	This guidance leaflet describes some potential service routes for the patient, resources (such as templates for anxiety treatment), inclusion and exclusion criteria for patients and overall aspects that practitioners should consider (such as collegial support and collaborating with others).
3	Treatment contract and TADA info	TADA	Service aid	The treatment contract supports joint relationships and collaborative work in restoring the oral health of patients.
4	Treatment plan	TADA	Service aid	The treatment plan is a template and outline for each session and describes the small and large goals intended for the patient to achieve throughout the service pathway.
5	Coping plan	TADA	Service aid	This coping plan is jointly completed by the patient and TADA dental practitioner. The coping plan aims to aid in the dental restoration phase, making the patient and the follow-up dental practitioner aware of their anxiety triggers, warnings and the need for adjustment.
6	*Patient Handbook*	TADA, 2019	Guidebook	Patients receive a handbook describing the aim and outline of the service. The handbook includes details on anxiety and trauma and the effects they have on the dental setting.
7	White Paper 35 ‘Accessibility, Expertise, and Social Equalisation in the Future Dental Health Service’	Ministry of Health and Care Services, 2006–2007	Policy paper	Describes the government’s objective to create and offer equal health care services regardless of diagnosis, place of residence, personal finances, gender, ethnic background and individual life circumstances.
8	‘Facilitated Dental Health Services for People Who Have Been Subjected to Torture, Abuse or Odontophobia’	The Norwegian Directorate of Health, October 2010	Report	The first report developed prior to TADA teams being established. This report provides a description of different aspects of the patients and the rationale for why they need facilitated dental treatment or therapy.
9	Job description: Dentist/dental hygienist	TADA	Role description	This job description describes the expected tasks that a dental practitioner should execute.
10	Job description: Dental assistant	TADA	Role description	This job description describes the expected tasks that a dental assistant should execute.
11	Job description: Psychologist	TADA	Role description	This job description describes the expected tasks that a psychologist should execute.
12	‘TADA Survey’	Simonsen, Ø., 2019	Survey	This is a survey conducted by a private dentist (not a TADA service practitioner) who collected thoughts from other (mostly private) practitioners regarding the TADA service. Thirty statements were reported, all of which voiced negative concerns about the workings of the service.
13	‘Overall Reporting on the TADA Service’	The Norwegian Directorate of Health, 2016, 2017, 2018, 2019	Report	Yearly reporting of data on the types of patients enrolled in the service, waiting lists, the total number of TADA teams within each county and the economy of the service.

#### Interviews

The first author emersed herself in the research context by participating in regional and national TADA service meetings, network gatherings and by shadowing TADA practitioners. Throughout this work, she made memos from meetings and kept a reflective journal [44]. The first author has master’s-level qualitative interview experience and training.

The sample interviewed comprised professionals responsible for the national and regional development of the TADA service. The TADA service is stakeholder-driven. This means that service developers who initially targeted the patient population, also participated in designing and implementing the service and had various roles in its delivery.

First, key informants were identified in the main service document (Table 2, document number 8) [9] and were purposively recruited based on their role, i.e., their primary involvement in, and responsibility for, the initial service design. As the odontology field within Norway is relatively small, further recruitment occurred through a snowballing strategy [45, 46]. Participants were invited to participate through email.

A total of 14 informants were identified as primary stakeholders who had a direct impact on the TADA service design and rationale. Of these informants, two had retired, but the remaining 12 agreed to participate in the interviews. As two participated in the same interview, a total of 11 interviews were ultimately conducted. As mentioned above, the TADA service is stakeholder-led and implemented. This means that the recruited informants had played key roles in identifying the need for the service, conceiving and implementing the service and acting as service deliverers. At the time the interviews took place, 10 of the 12 informants were also practising service deliverers (either dental practitioners or psychologists). The remaining two stakeholders were members of the dentistry profession but were currently acting as managerial staff. Their role was to oversee the national structure of the TADA service, develop national service guidelines and cultivate cross-sectional learning.

All interviews were audiotaped and transcribed verbatim immediately after they were completed. Stakeholders chose to proceed with interviews at their own clinics or workplaces. Other than the lead author and interviewees, no one was present during the interviews. The average duration of the interviews was 54 minutes. Consistent with the realist perspective, preliminary theories that emerged from the document analysis served as the basis for the initial interviews, the content of which was then used to structure subsequent interviews. Realist interviews seek to identify, explore and refine emergent theories regarding the workings of a given phenomenon – in this case, the TADA service/programme [47]. TADA stakeholders were thus asked to answer questions related to their experiences with and rationale for conceiving, designing, structuring and delivering the TADA service.

Individual, semi-structured interviews were conducted. While focus group discussions may have generated rich data, individual interviews were chosen over focus group interviews due to the geographical distance between the stakeholders participating in the project. In addition, discussions of work practices and vulnerable patients were anticipated to potentially provide detailed and sensitive information, and hence, individual interviews were deemed more fit for purpose [48, 49]. The schedule was semi-structured in nature, which allowed new ideas to be brought up during the interviews, underpinning the explorative approach of this study [50].

Voluntary participation was based on informed consent. Written consent was obtained from the study participants. The Norwegian National Centre of Research Data (NSD) evaluates how to manage and protect data in research projects in Norway ethically. The NSD committee approved this study’s data management and handling (Project No: 619754). Due to the nature of this study, not collecting data from vulnerable patients or health information, there was no need for further evaluation by the Norwegian Committee for Medical and Health Research Ethics.

### Analyses and data management

**Fig. 1:**
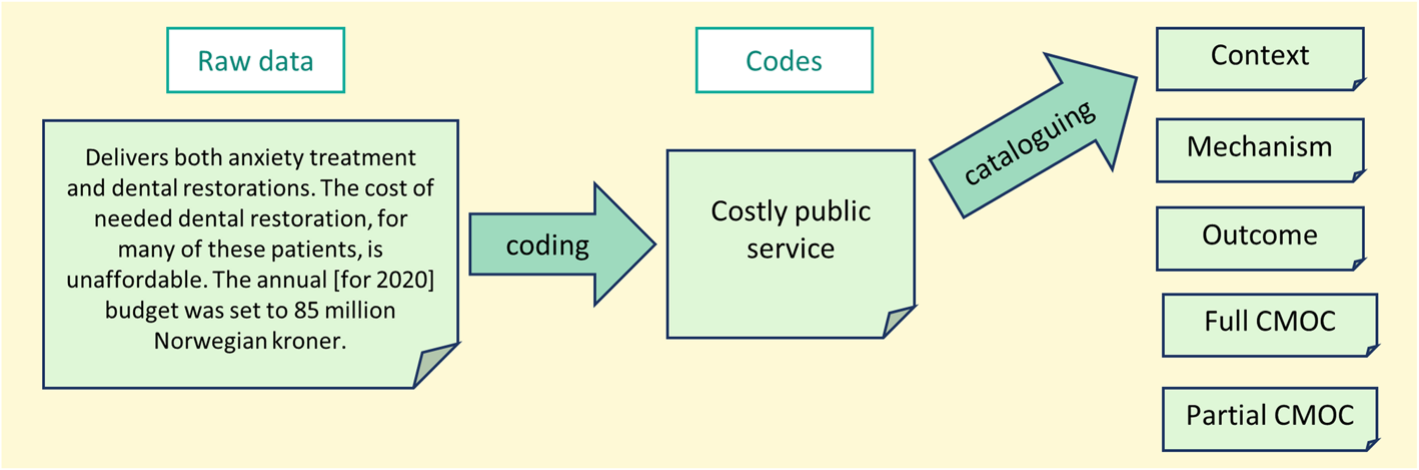
Steps of coding and cataloguing. The figure shows the steps of coding and cataloguing using an example of text from a policy document

The unit of analysis in realist evaluations are programme theories (Table 1), generated from the specific analytical steps outlined below and as displayed in Fig. 1.

#### Coding

The first step in the analysis involved open coding. The coding procedure consisted of reading the data material multiple times to ensure the first author had a comprehensive understanding before assigning a code name to each portion of the text, with each code corresponding to some aspect of the data, for example, *costly public service.*

#### Cataloguing

Codes are then reviewed for the purpose of relating them to an area of insight. Once the codes had been related to insights concerning contexts, mechanisms or outcomes (in isolation) with respect to the CMOC as a unit, they were definitively catalogued. For example, the code *costly public service* was catalogued under *context* (see Fig. 1).

#### Configuring

Understanding the causal links between contexts, mechanisms, and outcomes is central to a realist evaluation. Therefore, identifying the exact CMOC is an important part of the analysis. The CMOC was determined by first identifying the service outcome, which was then used as a guide to iteratively review the data material until the causal mechanisms underlying the outcome were understood. The analysis was directed by *theorising* how this outcome came about (mechanisms) and what about the TADA service generated this outcome (context). Generating this CMOC was as retroductive as it was iterative; that is, the CMOC revealed the interaction and generative association between the context and the mechanism, in turn, leading to the outcome. This in turn, ultimately illuminated the essential connections between the individual components of the CMOC. The first author performed coding, cataloguing and configuring in this study. The trustworthiness of this preliminary analysis was confirmed through discussions of the outcomes of these processes with other authors of the paper, and codes, categories and configuration descriptions were adjusted following discussions.

Another central tenet of realist evaluations is understanding the architecture of the phenomenon. In this study, this meant comprehending how stakeholders designed the service, structurally speaking, to deliver the intended service outcomes. By conceptualising the architecture of the TADA service, its service pathways and delivery roles were also revealed.

For transparency and credibility, quotes are included in the Results section. These quotes were translated by the first author from Norwegian into English and then translated back into Norwegian from English by an independent party. Informants have been kept anonymous, quotes being marked by the profession of the informant (i.e., whether the service practitioner specialises in psychology or dentistry). For confirmability, the third author, who, at the time, was an active TADA practitioner responsible for developing and delivering the CBT training curriculum, member-checked the programme theories and the architectural understanding as this emerged. The qualitative software programme NVivo was used to manage the data [22, 51, 52].

## Results

Data analyses revealed four CMOCs describing the structural elements of the TADA service at the macro and meso levels, each of which affected the service outcomes. The micro-level data have been presented elsewhere [20, 21].

**Fig. 2:**
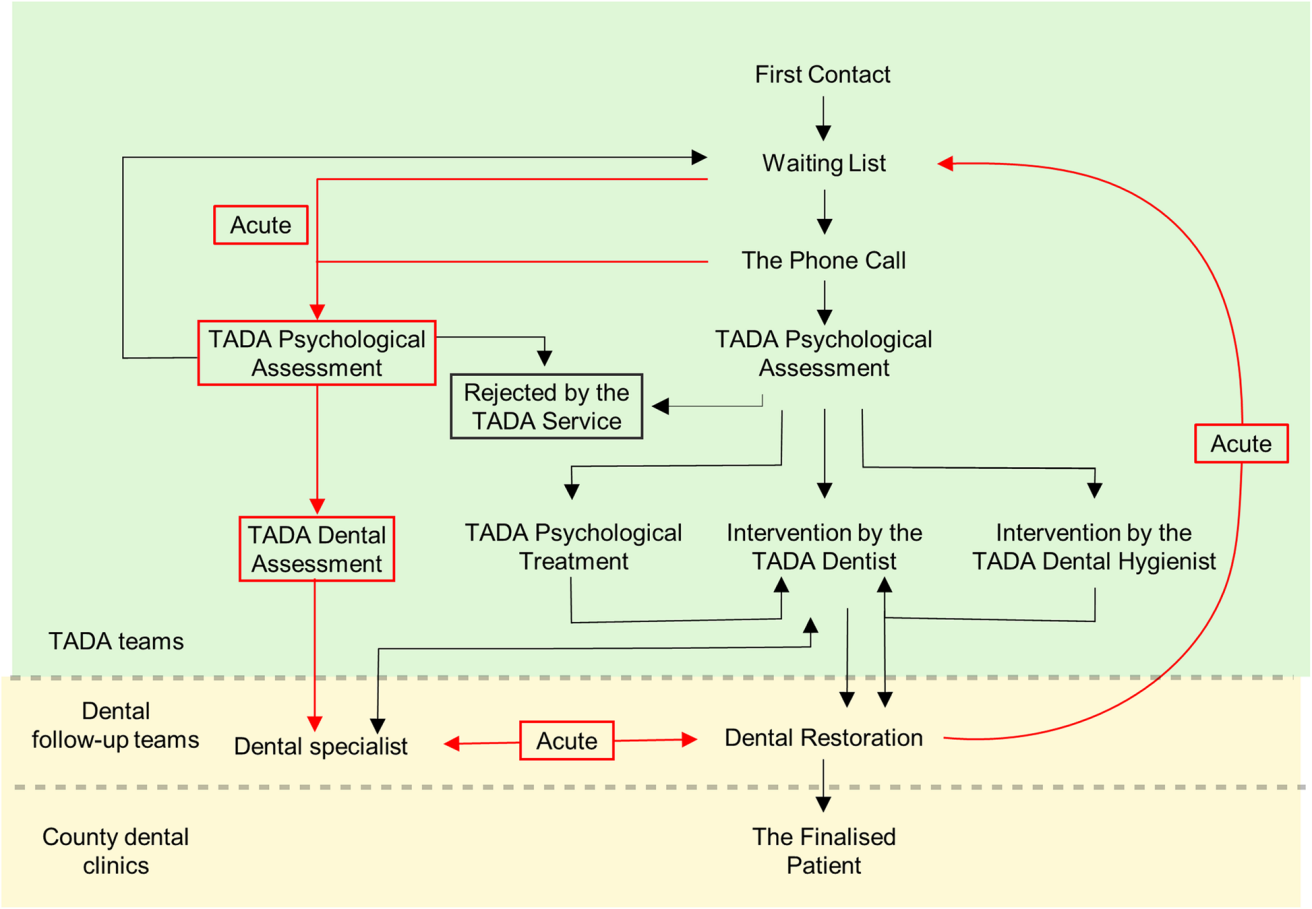
TADA service pathways

The TADA service aims to alleviate dental anxiety and restore oral health. The architecture of the TADA service, and the various pathways this promotes to achieve these end goals, – are presented in Fig. 2. Figure 2 also shows how the TADA service pursues an interdisciplinary approach by combining psychology and dentistry into a single service. Operationally, this means that the service is divided into two departments, each consisting of a separate team tasked with delivering a specific aspect of the service. The first department is staffed by an interdisciplinary team whose focus is on the psychological dimensions of the TADA service. Specifically, this team is charged with mitigating or alleviating trauma or anxiety among patients. The second department is focused on oral health, with its primary objective being the restoration and maintenance of patients’ oral health.

Figure 2 depicts the different pathways and processes patients may experience when navigating the architecture of the TADA service and who is responsible for delivering each stage of the service. First, patients initiate contact with the service, which places them on a waiting list. If the patients are in severe pain, they can call a phone number for an acute assessment. The pathway coloured in red indicates the acute route, which, depending on the patient’s needs, can provide oral restoration (typically with a general anaesthetic) before CBT. The pathways coloured in black indicate the more typical service routes, however. Once the waiting time for a patient, who did not need an acute assessment, is over, they receive a phone call from a TADA team deliverer to schedule an appointment with the TADA psychologist, who will assess the patient’s eligibility for service inclusion. Patients eligible for service inclusion are then given a referral to receive CBT from a dental practitioner or for additional psychological treatment before CBT if required. The TADA teams provide all the actions described above, which the figure depicts in the yellow section. Once the TADA team assesses the patient as having finished the CBT intervention, they move to the phase where their oral health can be restored. The dental restoration is provided by a dental follow-up team, as depicted in the green section of Fig. 2. After their oral health has been restored, county clinics, outside of the TADA service, are contacted to ensure that patients continue to pursue their oral health by regularly attending dental examinations with other services.

CBT is used to obtain a fuller understanding of the interconnections between cognition, behaviour and emotion, which can subsequently be challenged and resolved through a collaborative, active and direct approach [41]. A major feature of CBT involves exposing the patient to the causes or triggers of their anxiety. This occurs in TADA through *in vivo* exposure therapy in the dental setting. In the TADA service, dental practitioners perform this exposure therapy, which is intended to gradually but directly desensitise patients to the anxiety-provoking aspects of the dental experience [18]. The treatment duration is typically 12 sessions; however, field experience has shown that additional treatment sessions are often needed, with the number of sessions correlating to the severity of the trauma experienced by the patient. Any dental treatments administered by the psychologically oriented TADA team during exposure therapy aim to alleviate anxiety. It is not about restoring oral health at this stage. The purpose here is to motivate patients to continue to pursue and eventually achieve dental restoration via the dental follow-up team after their anxiety has been effectively managed.

Upon completing CBT, the dental follow-up team takes over and is tasked with restoring the patient’s oral health. The follow-up team’s primary responsibility is to restore the patient’s oral health to an *acceptable* standard. This means that the patient should experience no oral pain discomfort or diseases, have satisfactory functionality and they should be able to communicate and participate in social settings without experiencing oral pain or other complications [53]. In collaboration with the patient, the dental practitioner in the TADA team decides when this transition to the follow-up team occurs.

### Programme theories

We developed our programme theories based on causal chains between contexts, mechanisms and outcomes in the TADA service architecture, as presented in Table 3 below. There were four discrete CMOCs that emerged.

**Table 3:** Context-mechanisms-outcome configurations (CMOCs): The building blocks for programme theories

CMOC	Context	Mechanism	Outcome
Number 1, relates to programme theory 1	TADA is a state-funded service. It delivers both anxiety treatment and dental restoration. For many of these patients, the cost of dental restoration is unaffordable. The 2020 annual budget for the service was 85 million Norwegian kroners (around 8 million euros).	*Increased accessibility*: a vulnerable group can access services they would otherwise be unable to afford. This makes it easier for these patients to improve their oral health. *Altered focus*: The service shifts from private to public.	An immediate outcome for patients is increased accessibility to services and hence increased service uptake. There are ripple effects for patients including improved quality of life.
Number 2, relates to programme theory 2	National guidelines set by the Norwegian government are open to interpretation. Some patients are in difficult life situations and may not always benefit from the full CBT dimension of the TADA service. Patients are heterogeneous in character.	The TADA teams *tailor* their approach by learning what the patient needs and they search for local resources in order to meet these needs.	The service delivers treatment, improving the oral health of patients who follow the service pathway. Not all patients meet the clinical assessment criteria for of alleviation of dental anxiety.
Number 3, relates to programme theory 3	The Directorate of Health controls the service. There is a lack of common meeting arenas. There is a lack of explicit leadership and guidelines from the Directorate of Health. Poor communication exists across teams (nationally and regionally). County legislation affects service delivery.	TADA teams become *self-reliant* and *protectionist* in their work.	The individual TADA teams work cohesively as a team but separate from other teams in the region.
Number 4, relates to programme theory 4	There is an increased incidence and severity of torture methods in countries from which migrants have fled. Accounts from the Directorate of Health reveal that few torture survivors have applied for the TADA service. Teams have adjusted to accommodate patients more quickly. There is possibly a lack of sufficient advertisement for the TADA service. When fleeing from conflict areas and trying to resettle in a new country, individuals do not necessarily prioritise dental anxiety and/or dental restoration. This patient group undergoes a long asylum interview in which their backgrounds are checked, and they are asked to describe their torture experiences. Being asked to do so again may be exhausting.	The patients may find the asylum process *overwhelming*. Their dental health is *not prioritised* at this given time. Patients were *unaware* that such a service exists.	The service is unable to reach and accommodate patients who have suffered from torture.

### Programme theory 1: subsidising the TADA service means oral health becomes a public project and dental avoidance behaviours become a public health concern. This consequently improves patient access and service uptake

In Norway, patients over 20 years old are responsible for paying their dental care. In our research, both policy documentation and service developers indicated that the cost of dental treatment could be a prohibitive factor for these patient groups. Without assistance, these patients could be unable to afford the restoration of their oral health. Thus, through state subsidies and a bi-dimensional approach, the TADA service contributes to the accessibility of dental treatment for this populations. This addresses not only cost issues but also anxiety-related behavioural issues. Government subsidisation of the TADA service means a shift in the way oral health care is viewed. There is a shift from viewing dentistry as a private service provision to a public service. It places oral health in the foreground among public health concerns. As depicted in the first CMOC (Table 3), reports reveal a service uptake among patients who presumably would otherwise have avoided dental services. Furthermore, service developers’ interviews revealed that they believe service enrolment has ripple effects on these patients. Patients report that they feel more capable of re-engaging in society and describe components linked to an increased quality of life.

*It is clear that such a service like this, it’s costly, yeah … but it provides quality of life… It has large effects, then, for a lot of people.* (Interview 3, service developer within dentistry)

*I think that, when I see a bill of 200,000 [Norwegian kroner, equivalent to 20,000 euros], I think, ‘money well spent’. For there may be someone who gets back to work, one who manages to be a mother or father again and can live normally, as a normal human being – get their dignity back. So, I’m pretty sure that Norway will get the TADA money back in, yeah ... with good returns later, not that year, but in a few years. I’m pretty sure it will earn itself back.* (Interview 10, service developer within psychology)

*I think that the TADA service has succeeded in helping many people. Oh lord, compared to when I started … a lot of people get help. It’s been on the agenda; everyone knows about it. This is not a thing the dentists can push away anymore, sweep away. So, the project is successful, I think.* (Interview 8, service developer within dentistry)

### Programme theory 2: catering to a heterogeneous patient group means adapting and tailoring the service to regional resources and patient requirements

The service policy directed by the Norwegian Health Directorate allows a degree of regional autonomy and professional interpretation regarding *how* practitioners should deliver the service. The patient group is described as heterogeneous (suffering from dental phobia or different types of traumas resulting from torture or abuse) and is complicated in terms of socioeconomic status, health and other life circumstances. Service developers, therefore, viewed a *standardised* service as inadequate and instead adapted the service in light of local resources and patient requirements. Local resources may include the use of anaesthesia and the incorporation of additional psychological therapy but may also entail *pausing* the patient during the service pathway. Pausing the patient means postponing therapy or dental restoration until the patient considers themselves ready to proceed. By adapting the service to local resources, TADA practitioners can tailor the service according to what it can realistically offer and what the patient can realistically achieve. This implies that some patients may receive complete dental restoration without meeting clinical outcomes of alleviated dental anxiety. Nonetheless, by being flexible and tailoring services according to available local resources and realistic goals, TADA practitioners can improve their patients’ oral health. In their own words, practitioners believe in finding the service pathway best suited to the needs and capacities of each patient.

*They are difficult to catch because they often have comorbidities; they have other issues in their life. And we have patients that I have given anaesthetics to [for dental restoration] and put on hold, because – I remember clearly a young lady, like 27 to 28 years [old], child welfare services were at the door, new little girl, little daughter, beaten by the partner, had a security alarm and … lived what you would call a difficult upbringing. Of course, she was being assessed for PTSD … and then she started feeling pain in her teeth, so she felt she needed to deal with that, and for her to come once a week for CBT and twice a week for PTSD treatment and meet with child welfare services and no […] It became too much for her.* (Interview 3, service developer within dentistry)

*What is being successful for the patients? […] We have limited the goal. Previously, the goal was to go to the dentist regularly, but now we have seen that it’s too much for her in this round, so then we have said that the goal for this round is to have a clinical examination so that she can receive treatment under anaesthesia if necessary. […] but sometimes we just have to, we just have to adjust the goals a bit. If people are traumatised and are not capable of carrying out the treatment with tartar and sprayer and drill and all of those.* (Interview 4, service developer within psychology)

### Programme theory 3: a national service, operated by individual satellites, leads to a lack of communication, nationally and regionally, and isolation of each service from others

The Norwegian Directorate of Health seeks to maintain a similar standard of service across the country. This is achieved nationally by controlling service guidelines, implementing regulations and providing policies. However, the management of local resources and logistics is the responsibility of each county and each TADA team. This county-level compartmentalisation creates divisions between regional teams and how they implement and interpret the expectations expressed by the Directorate of Health. This hampers the delivery of the same standard of service across the country. These divisions, coupled with the lack of mutual meeting arenas, absence of explicit leadership and gaps in ministry guidelines, foster team isolation and impede effective communication and collaboration between the different TADA teams.

As a result, individual TADA teams have become more self-reliant and efficient at resolving challenges with local solutions, which are often not shared with other teams. There is however little consensus, either regionally or nationally, on how to interpret national guidelines or how much each team should adapt the treatment to the different patients’ needs. Despite these limitations, and perhaps because of them, each TADA team has become more cohesive.

*I probably feel that we, to a high degree, have become satellites at each of our clinics who’ve developed our own way of working … And, maybe a bit too protectionist in that, and instead of using the days – the semi-annual meetings for something very constructive, it’s very quick for everyone to just sit down to tell how they are doing it. And we develop some solutions for things, because the guidelines are okay – but less specific […] And when there is no common location there are no common meetings between the psychologists; for example, there is no regular hospitality with each other, no common meeting points ... no line between us and management.* (Interview 11, service developer within psychology)

*The Directorate of Health wanted this particular service to be a public offer. In that it’s public, they would also be in control of it […] in that it would be an equal offer… and that people working with training … according to a plan, and that the plan was quality assured …* (Interview 7, service developer within dentistry)

*Internally, we are stuck, each in our own cave.* (Interview 4, service developer within psychology)

### Programme theory 4: lack of recruitment of torture survivors to the TADA service is explained by challenges that patients experience because of the migration process and poor dissemination practices

Although Norway is believed to host between 10,000 and 35,000 torture survivors, patient demographics from the TADA service show that only 21 patients were torture survivors during the period from 2012 to 2018. Several initiatives have been made to increase service access for this population, such as minimising the waiting time for admission and ensuring the presence of independent interpreters. Thus far, however, this approach has had a minimal effect. One explanation for this, according to stakeholders, is that torture survivors are already overwhelmed by the extensive paperwork they must complete when entering Norway, making registration for the TADA service an additional burden. Another potential explanation is that oral health for such patients is simply not a priority when compared to the challenges involved in resettling in a new country and attempting to fit into a new society.

Service developers considered a third possibility: patients may be unaware of the TADA service due to a lack of flyers, posters and other advertisements at asylum facilities or transcultural centres, and that word of mouth is just not effective at reaching this population. Simply put, the TADA service is currently unable to reach and treat torture survivors, one of its three target groups, and a possible explanation is that these patients may be overburdened or may not be aware that the service exists.

*A lot of them [asylum seekers] feel the TADA treatment is too extensive. They have to get to a certain place, so they would rather have treatment at a clinic that is near the school where they attend … where they are often attending introductory programmes … To be gone for a whole day, well, that can be difficult.[…] I definitely think they should have an offer, but I’m not really sure if … like, a lot of people have told me, ‘No, do I need to speak to a new psychologist? I have a psychologist. Can’t he/she do this?’* (Interview 1, service developer within dentistry)

*[The informant imitates a patient] ‘You know what, now I have finally got, after many years fleeing, then I finally got a residence permit, got myself a house, Norwegian, of course.’ – There is a lot to establish in Norway; to start such an anxiety treatment or dental treatment is not on the priority list. So, we have tried very hard to recruit them, despite persistent attempts to make a deal with the municipality about a two-week waiting list, and we have invited them to cooperation meetings.* (Interview 4, service developer within psychology)

## Discussion

This realist evaluation of the TADA service revealed that service developers are guided by four main programme theories explaining service outcomes at a structural level.

In our first programme theory, we describe how, in a context in which a service is state-subsidised, oral health shifts from being a private to a public concern. This results in an increased service uptake for TADA patients. Seen from a service perspective, this means that, by removing financial barriers, services like the TADA service hold the potential to reach a patient population that has otherwise been found to avoid dental services. Subsidising dental services and making oral health a public health concern has been the target of an ongoing debate in Norway. Currently, adults in Norway are expected to care for their oral health privately. The TADA service is an exception to the Norwegian norm by offering a bi-dimensional service that covers both psychological and dental needs free of charge.

Providing a subsidised service that tackles both psychological and dental needs implies that the Norwegian state addresses and acknowledges the severity of oral health neglect, as it now provides an equitable oral health policy at a societal level [54, 55]. Cost is only part of the complex issue of oral health inequality and by including oral health services as a government priority, the government acknowledges that oral diseases and achieving oral health is a critical aspect of a more holistic health picture [56, 57]. Oral health inequality is currently the subject of heated national debate due to the increasingly wide societal gaps between those who can afford dental care and those who cannot [54]. Consequently, when access to dental care is unequal, social inequality worsens, as only those able to afford such care will benefit from it [54, 58–61].

Further, patients may not be initially motivated or able to pay for psychological treatment that would enable them to access dental services more easily. In Norway for instance, studies have indicated patients’ lack of willingness to pay for dental care after receiving CBT, practising relaxation methods or receiving nitrous oxide sedation in the dental restoration process [62]. The results of this study revealed that patients were less willing to pay for such procedures pre-treatment (24%, *N* = 65) but were more willing to do so post-treatment (71%). Based on these findings, the authors argued that patients might avoid treatment unless or until it is subsidised [62]. This reflects international studies such as those described by Gulliford and colleagues [63] who discussed the meaning of health care access within the National Health Service in Britain and found financial barriers to be a significant deterrent to service utilisation and access for vulnerable patients [63, 64].

Programme Theory 1 also describes how, by providing a service through which patients can have both their psychological and oral needs met, service deliverers are able to observe how their patients are re-engaging in society and experiencing an improved quality of life. When patients have restored their oral health, their fear of ‘revealing’ their mouth [7, 65] diminishes, allowing them to re-engage and participate in society [65, 66]. This confirms research from countries like Germany, Sweden and Brazil who also link quality of life and dental fear, dental pain and dental access [66–68].

Our second programme theory revealed that service developers acknowledge that some methods will work for some patients, but not others, and that this depends largely on the local resources that a team can manage to acquire and on what patients realistically can achieve in light of their heterogeneity. There is a balance to be achieved between customising the service to meet individual patient needs and adhering to organisational standards. This is typical within health and welfare services and can be challenging for TADA teams. In their position, they are required to interpret organisational guidelines and practise professional discretion through direct contact with patients. For their part, the TADA teams have a sense of autonomy, as the TADA guidelines do not explicitly mandate how the service should meet its end goal of providing patients with an acceptable level of oral health. Thus, they are permitted to use their professional discretion when following the pathways depicted in Fig. 2. This means there is flexibility and room to adapt the TADA service to the specific patient’s needs and requirements. As the service is currently structured, the service deliverers can practise discretion, allowing them to customise the service to accommodate the patients’ needs without being overly constrained by the Directorate of Health’s top-down expectations. The TADA service’s approach to this reflects, what other authors have described, as a hybrid solution that incorporates both top-down and bottom-up approaches [69–71].

Our third programme theory examines how the TADA service is situated within a context in which there is a lack of joint meeting arenas and centralised leadership and where there is a certain ambiguity generated by county-level compartmentalisation and poor communication between teams. In such a context, our analyses revealed that teams are compelled to be more self-reliant and protectionist about their work, and as an outcome, they must operate in isolation. Therefore, as it presently stands, the structure of the service is not conducive to the cultivation of a national standardised service.

Nevertheless, working in isolation was also found to enhance a sense of team membership and, thus, foster greater cohesion. When teams share a set of assumptions and demonstrate like-minded thinking, this cohesion can be strengthened [72]. A strong team cohesion could be important, as this could translate to better performance, primarily because the team members can trust each other’s professional skills [73]. In other words, psychologists and dental practitioners can lean on each other’s discretion. Notwithstanding, as the contextual backdrop of the TADA service is devoid of national meeting arenas and bilateral meeting places, team-based solutions to service challenges typically remain within each team. This, in turn, prevents teams from learning from each other’s experiences, which is problematic given that local solutions and innovations could be beneficial for the TADA service nationally.

The research presented in our fourth programme theory specifically addresses torture survivors. We found that the TADA service is unable to accommodate torture survivors and that its current design appears to be ineffective at reaching and enrolling this target service population. Therefore, there could be a lack of understanding of the circumstances torture survivors find themselves in. This may explain the failure of the TADA service to reach this targeted patient population [74]. The literature on torture survivors is limited and focuses mostly on the quantitative reporting of their dental status, which is often poor [14, 38], thereby underscoring the need for the TADA service. One Australian study [75] found that barriers, such as long waiting lists and a lack of interpreter services, discourage torture survivors from enrolling in available dental services. However, programme theory 4 demonstrates that the TADA teams do adjust waiting times and invite independent interpreters to participate in the service but that despite this, the requirements for asylum seekers remains overly bureaucratic and often overwhelming for patients.

By studying the TADA service's contexts, mechanisms, and outcomes outlined above, we can better understand how the structural components of a dental anxiety service may address the needs of vulnerable patient groups. Based on these findings, we now provide specific recommendations for policymakers looking to implement similar services (Table 4).

**Table 4:** Recommendations based on our programme theories

Programme Theories	Recommendations
Programme theory 1: subsidising the TADA service means oral health becomes a public project and dental avoidance behaviours become a public health concern. This consequently improves patient access and service uptake	We recommend that policy makers consider public-subsidised anxiety treatment and dental services for patients with a history of torture or abuse or with dental phobia to promote successful service uptake and potentially impact these patients’ quality of life.
Programme theory 2: catering to a heterogeneous patient group means adapting and tailoring the service to regional resources and patient requirements	We recommend a hybrid bottom-up and top-down approach when designing dental services that address both the psychological and dental needs of vulnerable patients. A hybrid approach would allow the TADA team to interpret national guidelines, often set to meet a larger population, to match their local context. Allowing for this flexibility, means that service deliverers would be in a better position to use their professional discretion. National guidelines should be seen as service enablers rather than service constraints.
Programme theory 3: a national service, operated by individual satellites, leads to a lack of communication, nationally and regionally, and isolation of each service from others.	We recommend an increase of opportunities for regional TADA teams to meet. These events could range from annual service conferences, that encourage the exchange of local solutions, to interactive digital platforms on which cases could be easily shared and discussed. The latter is a timely option given the current COVID-19 pandemic.
Programme theory 4: lack of recruitment of torture survivors to the TADA service is explained by challenges that patients experience because of the migration process and poor dissemination practices	We recommend that service developers develop specific recruitment strategies for torture survivors, perhaps collaborating with institutions that process migrants and asylum seekers at their point of entry into Norway. These collaborations should seek to relieve first the administrative pressures currently placed on migrants and, secondly, clearly include their dental health needs in the entry process. Upon uptake, service deliverers, working in services such as TADA should be particularly cogent of the specific needs of torture survivors and their associated psychological and dental needs/treatment.

### Limitations and future directions

The study design took an exploratory approach to understand the context, mechanisms and outcomes of the TADA service. This has allowed us to come closer to answering why and how the TADA service works, for whom and under what circumstances. Therefore, the project has, in the Norwegian setting, presented an initial set of programme theories from the TADA service developers’ perspective to explain how services for patients with dental anxiety and/or trauma may function. The in-depth exploration of the perspectives of this group offers rich insight into how this setting is currently functioning.

However, we accept our limitations concerning the generalisability of the findings. The current sample size of 12 participants of this exploratory study could be considered small but as such is representative of the size of the small pool of stakeholders in the Norwegian setting who currently play an active role in service delivery regionally and nationally. Further, the TADA service is unique to the Norwegian setting, which compromises the transferability of our findings to the international arena, even where the need for services for patients with dental anxiety and/or trauma is shared. Nevertheless, we have maximised transferability through a detailed description of the TADA setting. To overcome our small sample size, we supplemented our data with policy documents relevant to answering our research question. We would recommend, however, that future research explore the programme theories held by service developers of similar services internationally and compare and contrast these with the ones we have found here in Norway. In realist evaluations, programme theories are constantly being refined. The theories developed from the individual interviews and documentary analysis conducted here could be enriched with the use of focus group interviews with the stakeholders, or multiple individual interviews, to give the stakeholders opportunity to refine their programme theories. However, 12 of the 14 stakeholders identified as key participants in our study were located across the country and had busy schedules. Thus, multiple interviews or focus groups at mutual arenas were currently difficult to orchestrate. If opportunities are provided for more collaboration events between services (Table 4), these opportunities may arise in the future. We also recommend that programme theories be further refined by including the patient perspective [20].

## Conclusion

The bi-dimensional structure of the TADA service, which provides both anxiety and dental treatment free of charge, has increased service uptake among a vulnerable patient population that otherwise tends to avoid dental services. Service developers believed that a hybrid bottom-up and top-down approach is beneficial and allows teams to practise professional discretion and tailoring the service to meet the needs of individual patients. Individual TADA teams are cohesive structures but there is a lack of regional or national consensus and communication. Lastly, there is an uneven uptake of the TADA service, with torture survivors apparently failing to take use of the service. Further research should look at refining the programme theories presented in this paper with patients themselves and supplementing the triangulatory data method, which could enhance the understanding of why and how the TADA service works, for whom and under which circumstances.

## Data Availability

The data material and analyses were written and conducted in Norwegian. Upon reasonable request, the data material will be translated and made available from the corresponding author.

## Abbreviations

TADA: torture, abuse and dental anxiety
CMOC: context + mechanism = outcome configuration
CBT: Cognitive behavioural therapy
PTSD: post-traumatic stress disorder
NSD: The Norwegian National Centre for Research Data
